# Commentary: Echocardiographic Evaluation of Hemodynamics in Neonates and Children

**DOI:** 10.3389/fped.2018.00076

**Published:** 2018-04-05

**Authors:** Yogen Singh

**Affiliations:** ^1^Department of Neonatology and Pediatric Cardiology, Cambridge University Hospitals NHS Foundation Trust, Cambridge, United Kingdom; ^2^University of Cambridge Clinical School of Medicine, Cambridge, United Kingdom

**Keywords:** hemodynamic evaluation, functional echocardiography, hemodynamic assessment in intensive care, echocardiography in NICU, neonates and children

The author and handling Editor declare a potential conflict of interest regarding their previous collaboration on the publication (1) which was brought to the attention of the editorial office post-publication. They apologize for not having declared this previously but state that this does not change the scientific conclusions of the article in any way.

## Author Contributions

The author confirms being the sole contributor of this work and approved it for publication.

## Conflict of Interest Statement

The author declares that the research was conducted in the absence of any commercial or financial relationships that could be construed as a potential conflict of interest.

## References

[1] de Boode WP Singh Y Gupta S Austin T Bohlin K Dempsey E Recommendations for neonatologist performed echocardiography in Europe: consensus statement endorsed by European Society for Paediatric Research (ESPR) and European Society for Neonatology (ESN). Pediatr Res (2016) 80:465–71. 10.1038/pr.2016.126 27384404 PMC5510288

